# HIV-1 Vaccine-elicited Antibodies Reverted to Their Inferred Naive Germline Reveal Associations between Binding Affinity and *in vivo* Activation

**DOI:** 10.1038/srep20987

**Published:** 2016-02-16

**Authors:** Kaifan Dai, Salar N Khan, Yimeng Wang, Linling He, Javier Guenaga, Jidnyasa Ingale, Christopher Sundling, Sijy O’Dell, Krisha McKee, Ganesh Phad, Martin Corcoran, Richard Wilson, John R Mascola, Jiang Zhu, Yuxing Li, Gunilla B Karlsson Hedestam, Richard T Wyatt

**Affiliations:** 1 IAVI Neutralizing Antibody Center at TSRI, La Jolla CA; 2 Department of Immunology and Microbial Science, The Scripps Research Institute, La Jolla CA; 3 The Scripps CHAVI-ID, The Scripps Research Institute, La Jolla CA; 4 Department of Microbiology, and Tumor Cell Biology Karolinska Institutet, Stockholm SE; 5 Vaccine Research Center, Bethesda MD.

## Abstract

The elicitation of HIV-1 broadly neutralizing antibodies following envelope glycoprotein (Env) vaccination is exceedingly difficult. Suboptimal engagement of naïve B cells is suggested to limit these low frequency events, especially at the conserved CD4bs. Here, we analyzed CD4bs-directed monoclonal antibodies (mAbs) elicited by YU2 gp140-foldon trimers in a non-human primate by selective sorting using CD4bs “knock out” trimers. Following two inoculations, the CD4bs-directed mAbs efficiently recognized the eliciting immunogen in their affinity-maturing state but did not recognize CD4bs-defective probes. We reverted these mAbs to their most likely inferred germline (igL) state, leaving the HCDR3 unaltered, to establish correlates of *in vitro* affinity to *in vivo* activation. Most igL-reverted mAbs bound the eliciting gp140 immunogen, indicating that CD4bs-directed B cells possessing reasonable affinity existed in the naïve repertoire. We detected relatively high affinities for the majority of the igL mAbs to gp120 and of Fabs to gp140, which, as expected, increased when the antibodies ‘matured’ following vaccination. Affinity increases were associated with slower off-rates as well as with acquisition of neutralizing capacity. These data reveal *in vitro* binding properties associated with *in vivo* activation that result in functional archiving of antigen-specific B cells elicited by a complex glycoprotein antigen following immunization.

The trimeric HIV-1 Env gp120 and gp41, mediate viral entry initially by binding to the primary receptor, CD4, and the co-receptor, CCR5^1^,^2^,^3^,^4^,^5^. These are the sole virally encoded proteins on the surface of the virus and are also the targets for neutralizing antibodies. These glycoproteins are of high interest as components of an effective vaccine, especially in regards to trimeric presentation of Env to the humoral immune system^6^,^7^,^8^,^9^. However, elicitation of broadly neutralizing antibodies (bNAbs) to diverse HIV-1 isolates has proven extremely difficult^6^,^10^,^11^. Such antibodies are elicited relatively infrequently in HIV-1-infected individuals, known as “elite neutralizers”, demonstrating that, under certain circumstances, the human immune system is capable of generating such broadly effective antibodies^12^,^13^,^14^.

One attractive target on Env is the conserved gp120 CD4 binding site (CD4bs), which interacts with the invariant primary viral receptor, CD4. This site is a known target for several distinct, potent and broadly neutralizing monoclonal antibodies (mAbs) isolated from chronically infected individuals^15^,^16^,^17^,^18^. Suboptimal engagement of B cell receptors (BCRs) in the naïve repertoire is suggested to be one potential limitation to the activation of broadly neutralizing CD4bs-directed B cells^19^,^20^,^21^,^22^,^23^. The prototypic CD4bs-directed bNAb is VRC01, heading the class of related bNAbs isolated from different HIV-infected individuals. Activation of the VRC01-class of CD4bs-directed antibodies can be enhanced in mice transgenic for germline-reverted VRC01-class mAbs by re-engineering the gp120:mAb interface^24^. These antibodies unusually use the HCDR2 of restricted VH1-02 or similar gene segments to bind gp120^25^. The germline versions of these gene segments are not known to be present in NHPs. Another class of CD4bs-directed bNAbs, use more classical HCDR3 interactions to recognize the conserved CD4bs neutralizing determinant^26^.

In this study we focused on the CD4bs, isolating a set of IgG-switched antibodies specific for this conserved neutralizing determinant elicited by gp140 trimeric model immunogens. We determined the germline origins of these mAbs to derive their most likely inferred unmutated germline (igL) state. Using the igLs as the most likely sequence of the BCRs on the naïve, CD4bs-directed B cells, we determined their range of affinities. Since we isolated the B cells from the resting memory compartment, these were CD4bs-directed B cells that possessed sufficient affinity for initial activation, then, allowing them to enter the germinal center (GC), exit the GC, and to be ultimately archived in the class-switched IgG compartment. Similar questions regarding B cell activation affinities have been addressed in mouse models with hapten^27^,^28^, but not in the context of a complex glycoprotein as done here.

In brief, the mAbs were isolated from an NHP inoculated with the YU2 Env immunogen, stabilized in a trimeric state with a heterologous foldon motif (gp140-F), following two inoculations so that we could study antibodies relatively close to their naïve, igL-state following *in vivo* activation. The foldon Env are trimeric, but are not of the more recent “well-ordered” class of spike mimetics, we and others have reported^9^,^29^. However, with foldon trimer-vaccinated animals already in hand, we were able to address such basic B cell biology questions using these trimers as model, multivalent glycoprotein immunogens. The experimental design of isolating B cells following just two inoculations attempts to minimize the likelihood that somatic hypermutation (SHM) had occurred in the mAb HCDR3 since this is difficult to unequivocally determine.

Following reversion of matched heavy and light chains to their igL state, we examined the mature and igL-reverted antibodies’ capacities to recognize the original eliciting trimer immunogen. Six of seven mAbs were able to efficiently recognize gp140 and gp120 in both their mature and igL states. We established a range of *in vitro* affinities for the igL reverted versions which were of relatively high affinity, between 2–150 nanomolar, establishing *in vitro* correlates with *in vivo* activation of these mAbs. Examination of additional related sibling B cells, isolated similarly, revealed detectable, but limited, HCDR3 mutations. Since igL lineage relationships are difficult to assign, we also tested these variants for binding. The igL mAbs derived from sibling B cells also all bound with detectable affinity. The affinities of the mature Abs, as expected, were higher and displayed detectable HIV-1 neutralizing activity. Our results suggest that B cells capable of recognizing the CD4bs with relatively high affinity exist in the naïve repertoire, or such affinity is easily achieved by minimal SHM, with implications for understanding of the initial B cell activation steps responsible for vaccine-induced B cell responses to the highly conserved CD4bs of HIV-1.

## Results

### CD4bs-directed mAb sequence analysis, igL assignments and reversions

**Figure 1:**
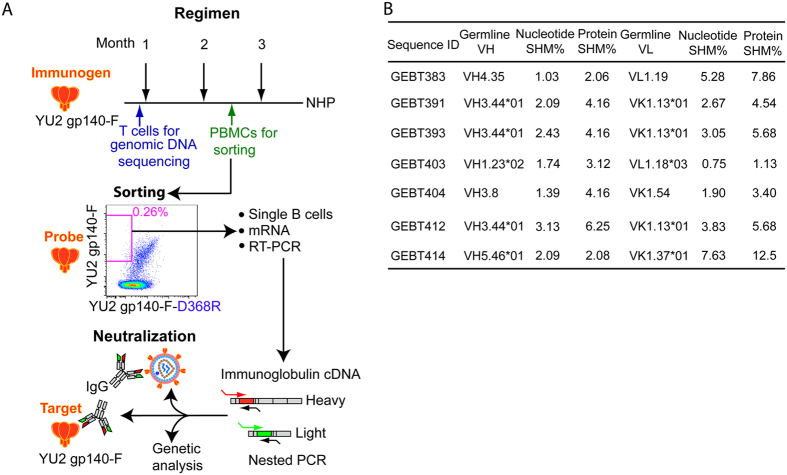
Schematic of immmunization regimen, mAb isolation and analysis “pipeline”. (**A**) Seven mAbs isolated following 2 Env trimer inoculations and resulting characterization at the genetic and protein level as shown. (**B**) Genetic analysis using the current NHP data base revealed mAb levels of SHM ranging from 0.75% to 7.63% at the nucleotide level as indicated and higher SHM when calculated at the amino acid level. New genes identified by genomic sequencing are labeled as ‘*’followed by numbers 01, 02 or 03.

We sought to determine recognition of HIV-1 CD4bs-directed mAbs against the matched eliciting immunogen in their mature and igL-reverted state following Env trimer vaccination. To begin with, following two vaccinations with the YU2 gp140-F immunogen in adjuvant, we isolated a set of CD4bs-directed antibodies from the memory B cell compartment of NHP F128 using a differential sorting strategy as previously described^30^ (Fig. 1). B cells were sorted using two fluorescently labeled trimeric gp140-F probes: gp140-F used to elicit the B cells and the isogenic trimer containing a mutation in the CD4bs, gp140-F-D368R, to allow isolation of CD4bs-directed single B cells. Following RT-PCR amplification of matched light and heavy chains from the putative CD4bs-directed mAbs, we cloned the heavy and light chain sequences into expression vectors for full reconstitution of the IgG by transient transfection as previously described^31^. Three of the VH and VL gene segments, when they were assigned to the most likely germline gene, did not efficiently recognize the eliciting YU2 gp140-F immunogen by ELISA. Therefore, suspecting that perhaps they were mis-assigned, we performed targeted genomic sequencing of all binding and non-binding HC and LC gene segments using the available published immunoglobulin sequence. We used the available data to generate site-specific 5′ and 3′ amplimers (Supplementary Table S1) as described previously^32^ and as described in Methods. The resulting TA clones from the targeted genomic sequencing from T cell-derived genomic DNA are shown (Supplementary Fig. S1).

Next, following the targeted genomic sequencing and, as well, inspection of the published NHP data base^30^, (Supplementary Table S1, Supplementary Fig. S1) we assigned all gene segments to known sequences or to the related, but new, gene segments identified by the targeted genomic sequencing. Newly identified gene segments were assigned a temporary designation of the closest match with a number variant label (see Fig. 1B). Following definitive identification of each gene segment, we determined the levels of SHM relative to the closest germline homologue. As expected, the percentage SHM was low, with the heavy and light chain sequences of mAbs isolated after two immunizations being relatively close to the assigned igL (Fig. 1B). The assignments of the seven mAbs aligned to the most homologous NHP heavy and light chains (HC and LC) are shown at the amino acid level (Supplementary Figs S2 and S3), with the deduced SHM changes highlighted below.

We next synthesized the appropriate heavy and light chain sequences using the following criteria to revert the heavy and light chains to their respective putative igL states. We back-reverted all deviations from gL that were at least two residues away from the HC V-D and D-J junctions to their nearest homologous rhesus gL gene segment. If there were deviations from gL at either junction, such that we could not definitively ascertain if the changes were N-nucleotide events in the original V-D-J Ig rearrangement or if they were somatic events, we made these variants and did not detect binding differences. Based upon the genetic analysis, we generated matching igL heavy and light chains of the CD4bs-directed mAbs and sub-cloned them into individual expression constructs for further investigation.

### ELISA binding analysis of the CD4bs-directed mAbs and their gL-reverted counterparts

**Figure 2:**
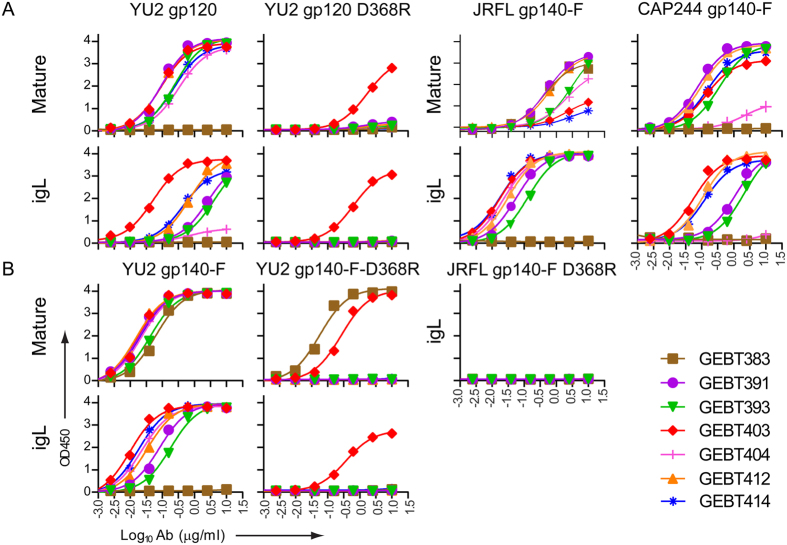
ELISA Env recognition properties of the mature and igL-reverted antibodies. (**A**) Binding of mature and igL IgG to soluble, monomeric YU2 gp120 (left) and isogenic gp120 possessing a non-conservative D368R mutation in the CD4bs (right) by ELISA. Mature and igL binding to JRFL gp140-F and CAP244 gp140-F are shown as two panels on the right. (**B**) Binding to clade B YU2 gp140-F trimers by the mature and igL mAbs (left) and to gp140-F D368R (right). Binding was abolished to D368R CD4bs “knockout” version of the YU2 gp140-F trimer (**A** and **B** panels on the left). The igL versions did not recognize the JRFL gp140-F D368R, consistent with naïve-state recognition of the CD4bs (**B**, panel on the right).

We expressed the mAbs and analyzed functional LC and HC association by SDS gels followed by Coomassie blue staining (Supplementary Fig. S4). To assess the capacity of the mature and igL mAbs to bind Env, we performed ELISA-based binding analysis to two forms of Env, monomeric gp120 and trimeric gp140-F. All the affinity-maturing, mAbs efficiently recognized monomeric gp120 (Fig. 2A) and all but one of the mature mAbs demonstrated marked reduction of binding to the gp120 D368R mutant. GEBT403 demonstrated reduced recognition of the D368R mutant, but not full loss of binding, similar to the control human CD4bs-directed broadly neutralizing mAb, VRC01 (Supplementary Fig. S5). Most of the fully reverted igL mAbs recognized monomeric gp120, but displayed lower levels of recognition, consistent with a lower affinity or avidity than the more affinity-matured antibodies.

We next performed a similar analysis using the eliciting trimeric immunogen, YU2 gp140-F, as the target antigen (Fig. 2B). All 7 of the NHP-derived mAbs efficiently recognized the eliciting gp140-F immunogen. We also assessed binding of the mAbs to gp140-F-D368R mutant trimer and observed that recognition was totally eliminated for most mAbs, and substantially reduced for the mature GEBT403. Also, GEBT383 was not sensitive to the gp140-F-D368R mutation, indicating that it was not a CD4bs-directed mAb as defined by our probe-determined differential sorting criteria. Instead, we were able to determine that GEBT383 recognized the His-tag present on the YU2 gp140-F immunogen (Supplementary Fig. S6). GEBT383 was not analyzed further on the basis that it was likely not CD4bs-directed. The partially D368R–sensitive, CD4bs-directed human mAb, VRC01, was used as a control and the glycan-directed human mAb, 2G12, was used as a normalizing mAb in each of the ELISA binding experiments (Supplementary Fig. S5).

To assess if the HC or LCs were most critical for igL mAb recognition, we generated mature, HC and LC igL chimeras. All chimeric mAbs tolerated the reversions in either the HC or the LC to produce stably associated heterodimers as analyzed by protein A affinity column chromatography, and all the mAbs generated had detectable matched HCs and LCs by SDS gel electrophoresis, indicating that the chimeric HC/LCs were functionally paired (Supplementary Fig. S4A Bottom panels). We performed ELISAs using chimeric versions of the mAbs with the eliciting gp140-F trimers as the target antigen. At the level of binding, there were mixed effects on recognition of env with either the igL-reverted LC or HC in the chimeric context. For example, in GEBT414, with igL HC, there was a complete loss of binding to gp140-F. (Supplementary Fig. S4B,C). This suggests that for optimal CD4bs-directed binding from the igL repertoire, at least *in vitro*, both the LC and the HC interactions are required for detectable apparent avidity-determined binding.

### Confirmation of CD4bs-directed specificity and intra- and inter- clade heterologous trimer recognition

We investigated the binding characteristics of the mAbs to other Env types. All mature CD4bs-directed mAbs efficiently recognized the clade B JRFL foldon trimers (JRFL gp140-F). All the igL mAbs also efficiently recognized JRFL gp140-F (Fig. 2A), but not JRFL gp140-F D368R (Fig. 2B). These data indicated that the igL B cell repertoire possesses CD4bs-directed specificities that exhibit some degree of recognition breadth. Five of the mAbs recognized the CAP244 gp140-F trimers both as mature and igL-reverted mAbs, consistent with the identification of BCRs displaying CD4bs-directed recognition breadth present in the naïve primate B cell repertoire (Fig. 2A).

### Binding kinetics comparing the affinity-matured, igL mAbs and Fab recognition of Env by Biolayer Light Interferometry (BLI) and Surface Plasmon Resonance (SPR)

**Figure 3:**
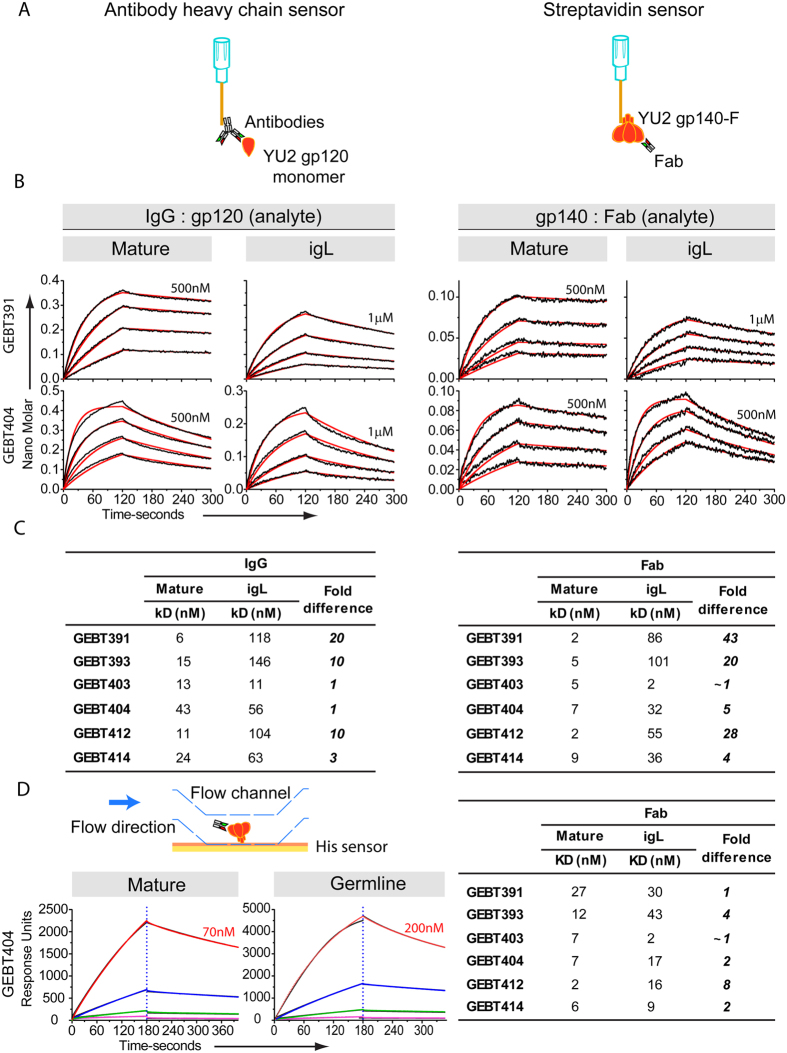
igL and mature antibody binding kinetics to gp120 monomer and gp140-F immunogen. (**A**) Schematic of the BLI assay formats. IgGs were captured on anti-human IgG Fc BLI biosensors and monomeric gp120, the analyte in solution and gp140-F was captured on streptavidin biosensors with the analyte in solution being Fabs. (**B**) Binding curves of selected igL IgG and their corresponding mature mAbs to SEC-purified monomeric YU2 gp120 (left) and to YU2 gp140-F (right). Data points are shown in black and the corresponding fits are shown in red. The Env concentrations used ranged from 500 nM to 1μM initial concentration as indicated above the highest concentration curve. 1:2 serial dilutions were used subsequently. Mature antibodies in general have higher affinity to gp120, displaying faster on-rates and slower off-rates. (**C**) Binding kinetics of the mature and igL IgG and Fabs to YU2 gp120 and YU2 gp140-F trimers respectively. (**D**) SPR binding kinetics were determined and used to derive affinities of both the mature and igL Fabs, with fold differences indicated (right), and the schematic of the method and binding curves are shown (left).

We next performed binding kinetics using the affinity-matured and igL mAbs to examine precise on/off rates and affinities of the mAbs to the CD4bs fully present on monomeric gp120. These data complement the binding ELISA results where avidity may contribute to mAb CD4bs-directed epitope recognition and precise binding constants cannot be derived. Accordingly, we performed BLI (Octet) binding analysis of the CD4bs-directed mAbs to size exclusion chromatography purified monomeric gp120 in solution to assess recognition parameters of both the mature and igL-reverted CD4b-directed mAbs (Fig. 3A left). Generally, the Octet binding kinetics was consistent with the ELISA data and representative examples of typical patterns are shown (Fig. 3B left, middle panel). Specifically, for the igL mAbs, most recognized monomeric gp120 with relatively high affinities, ranging from 11 to 146 nM (Fig. 3C, left). Increased affinity of most mature to igL pairs was detected even though only a few percent of SHM occurred following the two inoculations. Increased affinity of the mature mAb counterparts was most often associated with slower off-rates (Supplementary Figs S7 and S9A). Assessment of the chimeric mAbs by similar analyses, revealed light chain or heavy chain interactions that impacted on binding kinetics and affinities (Supplementary Figs S8 and S9A)

To determine absolute affinities to the eliciting trimeric gp140-F immunogen, rather than to the intact CD4bs present on monomeric gp120, we generated and purified recombinant Fabs of each of the six affinity-maturing and igL CD4bs-directed antibodies. We then performed Octet binding kinetics with the gp140-F trimers captured on the sensor surface and selected concentrations of the Fabs in solution as analytes (Fig. 3A, right). Similar binding patterns were detected for the igL and mature Fabs to the gp140 trimers compared to the IgG-to-gp120 analysis. Representative concentration-dependent curves and fittings are shown (Fig. 3B, right). Generally, affinities trended slightly higher for a given Fab compared to their IgG counterparts, but again the igL affinities were relatively high in their putative naïve state. Following the two gp140-F immunogen inoculations, affinities of the mAbs generally increased up to 10-fold compared to their igL counterparts, with the exception of the highest affinity mAb, GEBT403. Affinity for the gp140-F trimers was relatively unchanged for the mature Fab compared to igL-reverted GEBT403 Fab, the latter displaying exceptionally high-affinity in its igL state. For the other Fabs, affinities increased 4- to over 40-fold for the affinity-matured Fabs compared to their respective igL-reverted counterparts (Fig. 3C, right). Generally, the increases in affinity were accounted for by slower off-rates, indicating that this was an important factor in affinity maturation as previously described^33^ and in contrast to other studies where on-rate was associated with increased affinity^34^,^35^.

Since the affinities of the CD4bs-directed igL mAbs were relatively high as determined by Octet, we confirmed binding kinetics by surface plasmon resonance (SPR). We detected similar affinities compared to BLI of both mature and igL-reverted Fabs to the gp140-F trimers His-captured on the SPR chip surface. Representative concentration binding kinetic curves, fittings (Fig. 3D, left) and determined affinities are shown (Fig. 3D, right) confirming the relatively high affinities of the igL Fabs by this second biophysical method (Supplementary Fig. S9B).

### Analysis of sibling CD4bs-directed B cells

**Figure 4:**
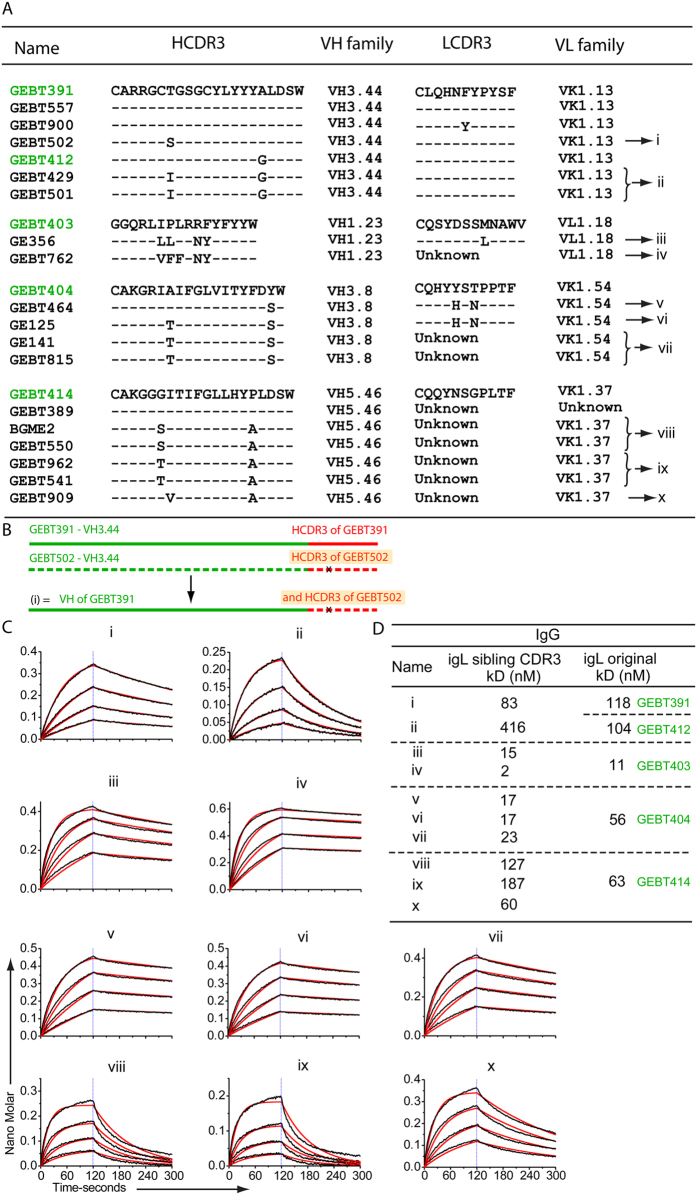
igL antibodies derived from sibling CDR3s. The CDR3 regions of the HC and the LC of the original igL versions (in green) are shown at the top for each gene family. The CDR3 regions of the siblings isolated are shown below with amino acid differences indicated. Sibling igL versions are labelled (i)–(x) (**A**). A schematic alignment depicting one representative sibling igL version as made is shown (**B**). Binding curves and fits of sibling igL binding to SEC purified monomeric gp120 by Octet (**C**). The kD values of the sibling igLs compared to the original igLs are shown (**D**).

To better determine if we had derived the correct igL assignments, especially the HCDR3s of the CD4bs-directed mAbs, we performed a second CD4bs-specific sort of the NHP F128 IgG B cells. Based on HCDR3 length and homology, as well as by use of the same V and J germlines, we identified related sibling clones. We noticed that there were occasional examples where one or two residues differed in the HCDR3 loop, making igL identification in the HCDR less certain as one of the siblings would have had to undergo limited SHM in the HCDR3 to account for these differences. Since it is then possible that the HCDR3 from either of the siblings could have been the one present on the naïve B cell, and represent the bona fide igL, we made versions of both sibling antibodies (Fig. 4A,B). In some cases, matching light chains were not amplified, so we used the existing LC. Upon subjecting the isolated sibling antibodies to BLI analysis by Octet, we determined that the binding affinities are not markedly different for many of the antibodies (GEBT391, GEBT 403, and GEBT404). For some antibodies, (GEBT 412 and GEBT 414) the newer versions possessed faster off-rates (Fig. 4C,D), suggesting that perhaps these siblings could have been the original igL on the naïve receptor. In any case, the number of changes in the HCDR3 is small, and does not change our conclusions as all of the mAbs bound with reasonable affinity to the gp140 trimer immunogens.

In an attempt to more conclusively identify the igL of each mAb, we sought to detect the putative ancestral CD4bs-directed mAbs in the naïve repertoire of NHP F128. We first interrogated the IgM B cell repertoire generated both prior to Env inoculation and the IgG class-switched B cell memory compartment during the Env-trimer immunization process. To amplify the naïve Ig repertoire, we isolated the mRNA from bulk PBMCs derived from this animal prior to Env trimer inoculation, generated a cDNA library using unbiased 5′RACE. We employed next-generation sequencing (NGS) to investigate the repertoire diversity. We visualized the heavy and light chain repertoires using GEBT391 and GEBT412 sequences as template by 2D contour plots (Supplementary Fig. S10A), but we could not detect related sequences in the IgM compartment. These results may be expected due to either the high turnover rate of naïve B cells or the small sample size or both. We then similarly interrogated the repertoire following 3 immunizations for related sequences. Among the IgG transcripts isolated from these “post-3” PBMCs, there were sequences that converged close to the query sequences of two CD4bs-directed mAbs (GEBT 391 and 412), along with the non-CD4bs-directed mAb, GEBT383. We could not detect related sequences for the other 4 mAbs. Closely related somatic variants characterized by an identity of 95–100% relative to the respective template antibodies can be seen on the identity/divergence plots (Supplementary Fig. S10A), indicating that these mAb lineages were detectable in the class-switched B cell compartment. Alignment and inspection indicated that we detected 11 related VH sequences to the GEBT391 query sequence and four to the GEBT412 sequence. Of all potential GEBT391 sibling VH sequences, there were mutations outside of the HCDR3, but none within the HCDR3. However, the HCDR3 similarity of GEBT391 and GEBT412 mAbs indicated that they themselves derived from a common B cell precursor, because they possessed only one residue difference between their respective HCDR3s, a relatively conservative A to G change at the base of the HCDR3. Since this is near the D-J junction, it is not clear if this is SHM-mediated or N-nucleotide related, but since all other sibling criteria are met, this is likely due to SHM (or PCR error). In any case, since both of these mature and igL mAbs bound Env with similar affinities (see Fig. 3C), this potential single SHM event does not change the affinity relationships with the known *in vivo* B cell activation/maturation events of focus here. For the non-CD4bs-directed mAb, GEBT383 there were many related sequences identified by deep sequencing, with no changes in the HCDR3 (Supplementary Fig. S11), consistent with the assertion that, in some cases, we can identify the bona fide BCR present on the naïve B cell prior to engagement and activation by the YU2 gp140-F immunogen by the methods described here.

### HCDR3 residues are critical for igL CD4bs binding

**Figure 5:**
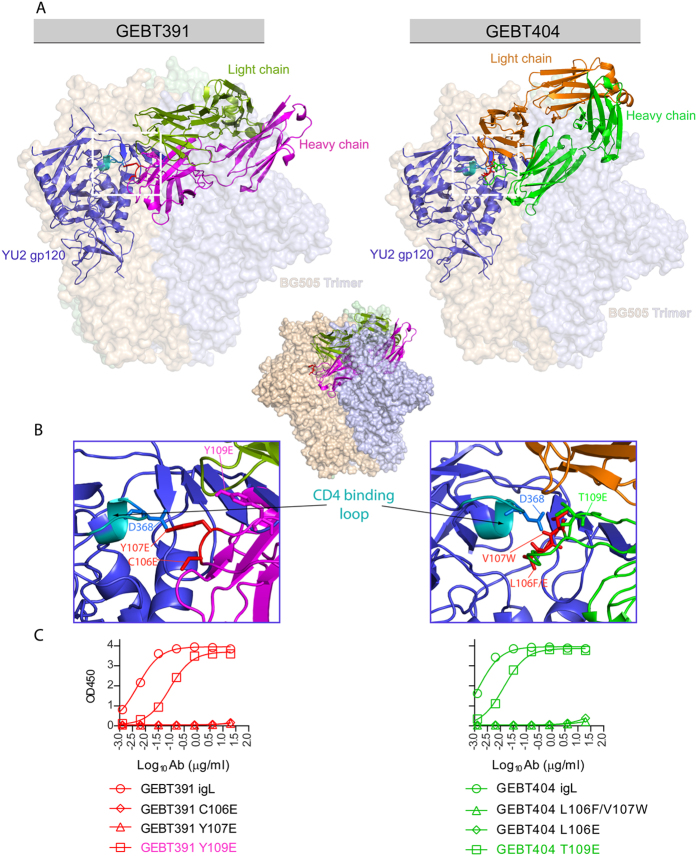
Binding properties of selected gL-reverted mAbs is HCDR3-dependent Residues in the HCDR3 region of two selected igL binders were mutated in specific sites within their respective HCDR3s to asses binding to soluble YU2 gp140-F trimers. These mutations were guided by previous modeling information of mature GE136 and GE148 and their interactions with gp120. (**A**) Fv regions of GEBT391 and GEBT404 were generated by the **P**rotein **H**omology/Analog**y R**ecognition **E**ngine (Phyre) and the model was used in the computational protein docking program, ClusPro2 to generate models of GEBT391 (left) and GEBT404 (right) binding to gp120. The gp120 core is modeled inside the structure of BG505 SOSIP (PDB ID: 4TVP) to depict orientation in the context of the trimer. Both antibodies approach the gp120 CD4bs “from the side”, creating clash with the adjacent protomer in the trimer context. This clash is shown by the partial burial of the Hc within the protomer colored light blue (middle). The residues involved in recognition are depicted with a white dashed box (**A**) and are shown magnified (**B**). GEBT 391 residue Y107 and GEBT 404 residue V107 interact with D368 of gp120. (**C**) Mutating residues 106 and 107 in both mAbs eliminated recognition but changing the residue 109, which is relatively distal to the CD4 binding loop of gp120, resulted in only a partial reduction in binding.

Next, to assess if the most critical paratope residues for recognition were in the HCDR3 as we observed previously for similar gp140-F vaccine-elicited CD4bs-directed mAbs^30^,^36^, we mutated the HCDR3 region of the two most potent igL CD4bs-directed binders. Using both inspection of their respective HCDR3s, comparisons to the binding motifs of the previously isolated CD4bs-directed NHP mAbs^36^ and structural modeling using Phyre^37^ and ClusPro 2^38^, we generated docking pairs of GEBT391 and GEBT404 to core gp120. (see Methods and Fig. 5A,B). The docking to core is shown modeled into the SOSIP trimer density to provide context. Using these models, we mutated GEBT391 and GEBT404 at specific sites within their respective HCDR3s to determine the impact on binding to the YU2 gp140-F trimers. These mutations were guided by previous modeling information of the mature CD4bs-directed mAbs, GE136 and GE148^36^, which display a cationic signature and hydrophobic region in their interactions with the CD4bs. Similar to the previous criteria and binding analysis, we confirmed the HCDR3 recognition requirements of GEBT391 and GEBT404. Specifically, for GEBT391, we mutated residue C106, proximal to D368 in the gp120 CD4 loop (by modeling, Fig. 5B, left), to an E residue, to create an electrostatic clash by this non-conservative substitution. This change abolished binding by the mutant igL IgG, by ELISA (Fig. 5C, left). Similarly, we changed Y107 to an E (Fig. 5B, left), to also create a clash with the gp120 molecular surface within the CD4bs and detected a complete elimination of binding (Fig. 5C, left). For the GEBT404 mAb, an L106 to E substitution completely abrogated binding (Fig. 5B right) as did L106F/V107W double mutations. Consistent with the modeling, GEBT 391 residue Y109 and GEBT404 residue T109, more distal from the contact sites, had about a log decrease in binding when mutated to a non-conservative E residue (Fig. 5B,C) indicating that the residue at position 109 is not as critical for binding in these antibodies. The angle of approach of these mAbs to the gp120 CD4bs of the monomer is “from the side” in the context of the SOSIP trimer, which creates a clash with the adjacent protomer in the quaternary-packed spike (Fig. 5A, middle), consistent with the inability of these mAbs to neutralize tier 2 HIV-1 isolates. This angle of approach is possible in the more open conformation of the eliciting gp140-F immunogen as previously described^36^. The importance of the angle of approach is further highlighted when we analyzed binding of the mAbs to well-ordered native-like trimers that are faithful mimetics of trimers expressed on the virus. When native-like JRFL-SOSIP trimers were captured via their C-terminal His-tags to retain well-ordered quaternary packing, they were well-recognized by the CD4bs-directed bNAb, VRC01, but not by any of the CD4bs-directed NHP mature or igL mAbs (Fig. S12, right panels). In contrast, when the native-like trimers were directly adsorbed to the ELISA plate, which disrupts native-like quaternary packing, both the mature and igL CD4bs-directed NHP mAbs recognized the solid-phase disrupted trimers nearly as efficiently as did VRC01 (Fig. S12, left and bottom panels).

### Neutralization capacity correlates with higher affinity to Env

**Figure 6:**
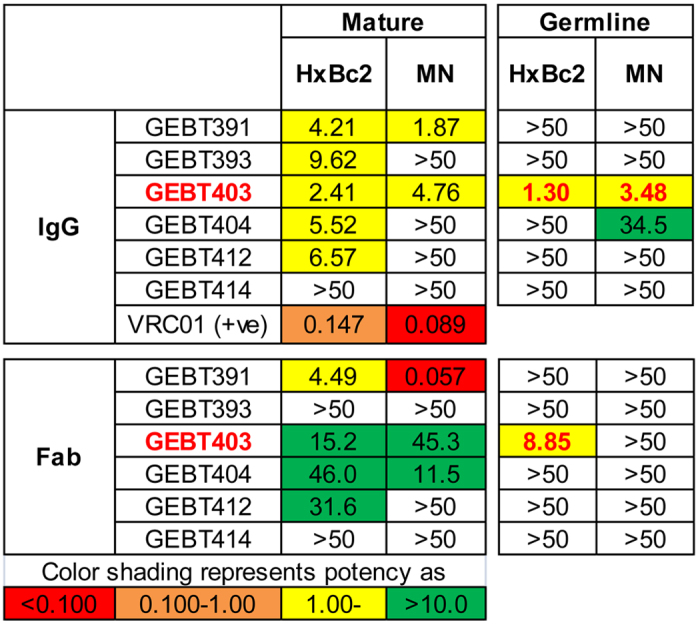
Neutralization of mature, chimeric and gL mAbs to HIV-1. Neutralization IC_80_ data of the mature mAbs (left) and gL-reverted mAbs (right) are shown and color-coded for concentrations regarding potency as indicated. Fab neutralization data and the IC_50_ values are tabulated in Fig. S12.

We then assessed the functional neutralization capacity of the six CD4bs-directed mAbs in their matured, chimeric versions and igL state against two viruses. We detected neutralization of the MN and HXBc2 viruses by all the CD4bs-directed mAbs (Fig. 6). Also a few of the chimeric antibodies neutralized each of these isolates (Supplementary Fig. S13). In contrast, none of the fully igL-reverted mAbs neutralized either of these neutralization-sensitive viruses, with the exception of the very high affinity igL mAb GEBT403, emphasizing the differential between high affinity binding and neutralization of even sensitive tier 1 viruses. We also assessed neutralization by the Fabs derived from the respective IgGs and observed that most of the mature Fabs neutralized HXBc2, albeit with reduced potency. However, only the high-affinity GEBT403 CD4bs-directed antibody neutralized HXBc2 as an igL Fab.

## Discussion

The NHP and human Ig loci display a high level of genetic similarity^39^,^40^,^41^,^42^ and NHPs have been used in numerous preclinical HIV vaccine studies and other disease models^43^,^44^. This experimental foundation allowed us to analyze NHP B cell responses directed at HIV Env trimers that have relevance to vaccine development in humans. In our current study, we report the isolation of seven new CD4bs-directed mAbs derived from NHPs following two inoculations of YU2 gp140-F trimers and we characterized their binding properties in both their affinity-matured and igL states. Following sequence analysis and identification of their most likely BCR progenitors, we reverted all seven of these mAbs to their igL state and analyzed their functional properties by several means of analysis. We demonstrated that by conventional ELISA, 6 of 7 igL-reverted mAbs recognized the known activating trimeric Env immunogen at the CD4bs. Recognition by the igL-reverted mAbs were shown to be CD4bs-specific, and most of the igL mAbs were capable of cross-clade recognition of a subtype C foldon trimer at the CD4bs. Binding kinetics implicated a relatively high threshold for *in vivo* B cell activation at the CD4bs for this set of mAbs. Finally, we demonstrate that nanomolar trimer binding affinity to the conserved Env CD4bs was encoded in the igL state without the need for affinity maturation of these mAbs, while some degree of affinity maturation was required for all but one mAb for detectable virus neutralizing capacity.

Studies of the origin and evolution bNAbs that arise as a consequence of several years of chronic HIV-1 infection are complicated by the fact that it is extremely challenging to know the status of the BCR on the originally activated naïve B cell since the precise activating Env is often not definitively determined^45^,^46^,^47^. This is also true of influenza HA-directed mAbs, since adults often are multiply exposed by both infection and vaccination, possessing extensive HA-specific memory B cell repertoires. Similarly for RSV, most adults are not naïve to the F or G proteins present on the surface of this pervasive respiratory virus^48^,^49^. A major advantage of the study design used here is that the eliciting gp140-F immunogen is well defined, as is the regimen, the immunization kinetics and route of administration in these well-controlled primate immunogenicity experiments. This provides us with a system that is well suited to address issues of igL engagement and affinity maturation. We also employed state-of-the-art next generation sequencing and 5′RACE IgG repertoire analysis to study the HCDR3s of clonal relatives of selected mAbs to ensure that the HCDR3 does not change much over time which indicates that the igL versions we synthesized are very close to the naïve state receptor that interacted with the vaccine immunogen. We were able to define a range of igL affinities that correlated with *in vivo* activation, results that have broad implications for our understanding of how the primate immune system responds to and matures following exposure to foreign protein antigens. Interestingly, we observed several antibodies that bound to the trimer in their igL state with reasonable affinity; however, the igL-reverted mAbs did not neutralize virus, except in one case where the initial igL affinity was in the low nanomolar range. Interestingly, low levels of affinity maturation and modest increases in anti-trimer affinity resulted in mAbs that gained the capacity to neutralize selected HIV-1 isolates.

In general, these data indicate that standard binding assays such as ELISA or Octet are relevant to affinity requirements for *in vivo* BCR engagement and B cell activation, at least for commonly isolated, CD4bs-specific B cells isolated following two inoculations of immunogen as described here. These results do not preclude that other Env-specific B cells become activated and enter the GC following vaccination, however, such events were not detected in the ag-specific set of mAbs described here. It might be that these are high avidity clones that efficiently form their “own” monoclonal GC within the secondary lymphoid tissues as described by Victora and colleagues, portending efficient seeding of the memory B cell compartment^50^. That recognition of the CD4bs exists in the naïve primate repertoire, and that limited SHM is required to gain the capacity to neutralize HIV-1 tier 1 isolates, may bode well for the use of the new, well-ordered SOSIP or native flexibly linked (NFL) Env trimers as immunogens^9^. We note that the CD4bs on these well-folded trimers is not recognized by non-broadly neutralizing CD4bs-directed antibodies such as those described here. However, if naive B cells do exist bearing BCRs that can recognize the CD4bs or other bNAb epitopes of these new trimers, relatively minimal affinity maturation may be sufficient to achieve neutralizing capacity of more resistant HIV-1 tier 2 strains

## Materials and Methods

### Ethics statement

The rhesus macaque (Macaca mulatta) of Chinese origin, designated F128, sampled for this study was described previously^51^. The Astrid Fagraeus Laboratory at the Swedish Institute for Infectious Disease control housed the animals. Housing and care was in compliance with the provisions and general guidelines of the Swedish Board of Agriculture, which are stricter than the European Directive 2010/63/EU on the protection of animals used for the scientific purposes. The facility was assigned an Animal Welfare Assurance number by the Office of Laboratory Animal Welfare (OLAW) at the National Institute of Health. All procedures were approved by the Local Ethical Committee on Animal Experiments (Stockholms Norra Djurförsöksetiska Nämnd) (ethical permit number N85/09 and N32/12). The methods were carried out in accordance with the approved guidelines. The animals were housed in rooms having daylight, in pairs in 4 m^3^ cages. The rooms are enriched with items that allow them to express their normal behaviors, such as comfortable bedding (wood chips, straw, hay, shredded paper, etc) in an area in which they can also forage for seeds, rice etc. Enrichment devices and puzzles are added/removed in addition to destructible toys to keep them entertained. They are offered different items such as fruit and vegetables to stimulate all their senses. They receive a bath at least once a week and have access to mirrors through which they can monitor the environment without having to resort to direct eye contact.

They were acclimatized to the housing conditions for about 6 weeks before the start of the experiment and subjected to positive reinforcement training to reduce stress. All immunizations and blood samplings were performed under sedation with 10 mg/kg ketamine i.m. (100 mg/ml Ketaminol; Intervet).

### Sampling

The immunization schedule of the experiment was described previously^51^. This consisted of gp140-F trimers in adjuvant (Abisco-100 with CpG-C) inoculated five times at monthly intervals. The antibodies described in the current paper were isolated from blood collected 1 week after the second vaccine inoculation.

### Isolation and production of NHP mAbs

NHP monoclonal antibodies were isolated by single cell sorting using flow cytometry (FACSAria, BD), followed by RT-PCR and amplification of VH and VL genes. Briefly, single CD4bs-specific memory B cells (CD3-, CD8-, Aqua Blue-, CD14-, CD20+, IgG+, CD27+, IgM-, gp140-F+, and gp140-F D368R-) were sorted into single wells of 96-well microtiter plates. The IgG matched LC and HC gene transcripts of the single NHP memory B cells were amplified by RT-PCR and cloned into eukaryotic expression vectors to produce full-length LC and HC ORFs. For expression of IgG, equal amounts of the LC and HC plasmid DNAs (25 mg each) were transfected with 0.1 ml of 293Fectin (Invitrogen) into 50 ml of FreeStyle 293 F cells at a density of 1.5 million cells/ml. Following expression, cell culture supernatants containing the associated LC and HC pairs as secreted IgG were harvested 5 days following transfection and purified by protein A Sepharose columns (GE Healthcare).

### Antibody sequence analysis and genomic assignments

The rhesus antibody sequence analysis and gL sequences have been described previously^30^. Briefly, IMGT/V-QUEST and JOINSOLVER was used to determine V(D)J gene family usage, while the specific V and J gene segments were annotated based on aligns with ClustalW to published rhesus germline databases^30^,^31^. The antibody alignments shown in the main and supplementary figures were generated by the sequence alignment editor and analysis program, BioEdit.

### Genomic DNA preparation, amplification, cloning and targeted genomic sequencing for germline gene segment identification

T cells derived from NHP F128 PBMCs were expanded with the T cell activation and expansion kit (Miltenyi Biotec). Biotinylated antibodies against non-human primate CD3 and human CD2 and CD28 were conjugated to anti-Biotin MACSiBead particles and were used to mimic antigen-presenting cells and activate resting T cells from peripheral blood mononuclear cells (PBMCs). T cells were expanded by adding IL-2 and fresh medium every 2–3 days. Genomic DNA was extracted using the QIAamp DNA blood kit (Qiagen). Flanking regions of the genes of interest were identified from ‘Ensembl’ genome database and primers were designed to amplify the VH regions of the genes by PCR (S1 Table). The amplifications were performed using a *Taq* DNA polymerase which adds a single deoxyadenosine to the 3′ end of the PCR product. Linearized vectors having complimentary 3´ deoxythymidine (T) residues allowed the VH gene amplifications to be ligated into the TA vector for subcloning (Life Technologies). The vectors were sequenced to identify the germline versions of the VH regions of the antibodies in this study. gL-reverted antibody constructs were then generated by synthesis after aligning antibodies LC and HCs to the corresponding germline V and J regions and reverting the nucleotide sequence to the putative gL configuration, while leaving the mature HCDR3 and LCDR3 unaltered.

### ELISA assay with purified protein

The IgG and Env glycoproteins (gp120 and gp140-F), which were produced in 293 F cells, were diluted in PBS (pH 7.4) at a concentration of 2 μg/ml and used to coat plates overnight at 4 °C. The plates were washed five times with 0.05% Tween 20 in PBS (PBS-T), blocked with 300 μl per well of blocking buffer (5% skim milk and 2% bovine albumin in PBS-T) for 1 hour at RT. 100 μl of each monoclonal antibody 5-fold serially diluted in blocking buffer was added and incubated for 1 hour at RT. Horseradish peroxidase (HRP)-conjugated goat anti-human IgG (H+L) antibody (Jackson ImmunoResearch Laboratories Inc., West Grove, PA) at 1:5,000 was added for 1 hour at RT. The plates were washed five times with PBS-Tween and then developed using 3,3´,5,5´-tetramethylbenzidine (TMB) (Kirkegaard & Perry Laboratories) at RT for 10 min. The reaction was stopped by the addition of 100 μl 1 N H_2_SO_4_ to each well. The readout was measured at a wavelength of 450 nm and all samples were analyzed in duplicate.

### Antibody binding kinetics analysis

The kinetics of mAb binding to YU2 gp120 and gp140-F, and Fab binding to gp140-F, were performed with an Octet RED96 system (ForteBio Inc, Menlo Park, CA) by BLI in a 96-well format. YU2 gp120 was subjected to size exclusion chromatography (SEC) to remove undesired oligomeric forms where applicable. The CD4bs-directed mAb-capture method was used to detect interaction with gp120. For the Fabs, the gp140 trimers where the trimers were captured by His tag and Fabs derived from the respective mAbs were in solution. Mature, gL and chimeric antibodies at 10 mg/ml diluted in PBS/0.2% Tween 20 were captured on the surface of the anti-human IgG Fc capture biosensors (ForteBio) for 1 min. After capture, a 1 min wash in buffer removed unbound Ab to establish a baseline signal. The biosensor tip was then immersed in wells containing monomeric gp120 in solution. The glycoproteins were two fold serially diluted from an initial starting concentration of 250 nM. Ab-Env associations (on-rate, K_on_) were measured over a 2 min interval, followed by immersing the sensors in wells containing buffer to measure dissociation (off-rate, K_dis_). KD values (in nanomolar units) were calculated as off-rate/on-rate (K_dis_/K_on_). The sensograms were corrected with the blank reference and fit with the software ForteBio Data Analysis 7 using a 1:1 binding model with the global fitting function (grouped by color, Rmax). The mature and igL Fabs were similarly analyzed by SPR with the YU2 gp140 captured on the surface of the chip by the C-terminal His tag.

### Ion Torrent PGM sequencing of NHP antibody libraries, bioinformatics pipeline and homology determination

The 5′-RACE protocol has been modified to improve the template preparation^52^. Briefly, total RNA was extracted from 10–20 million PBMCs with RNeasy Mini Kit (Qiagen). For unbiased antibody repertoire analysis, 5′-RACE was performed with SMARTer RACE cDNA Amplification Kit (ClonTech). The immunoglobulin PCRs were set up with Platinum *Taq* High-Fidelity DNA Polymerase (Life Technologies) with cDNA as template. The 5′-RACE primer contained a PGM P1 adaptor, while the reverse primer contained a PGM A adaptor. The primers each contained an appropriate adaptor sequence (A or P1) for subsequent PGM sequencing. 25 cycles of PCRs were performed and the expected PCR products (~600 bp) were gel purified (Qiagen). Sequencing was performed on the Ion Torrent PGM sequencer with the PGM™ Hi-Q 400 Kit using either an Ion 316 or 318 v2 chip for a total of 1200 nucleotide flows. Raw data was processed without the 3′-end trimming in base calling in order to extend the read length. The rhesus H, κ, and λ germline gene segments including the variable (V), diverse (D), and joining (J) segments were incorporated into the pipeline where such information is required for gene assignment (step 2), error correction (step 3), and determination of H/LCDR3 and variable region boundaries (step 5). For heavy chains, 65 V_H_ genes along with 60 D_H_ and 7 J_H_ genes were compiled into three libraries, while for light chains, 62 V_K_ genes along with 5 J_K_ genes and 50 V_L_ genes with 6 J_L_ genes were used in library construction, respectively. After full-length variable region sequences were obtained, a bioinformatics filter was applied to detect and remove erroneous sequences that may contain swapped gene segments due to PCR errors. To select heavy chain somatic variants, we identified heavy chains with an HCDR3 identity of 95% or greater to a given mAb.

### Neutralization assay

The assays were performed using pseudo-typed viruses that were produced by transient co-transfection of 293 T cells using the HIV-1 *env*-deleted backbone plasmid, pSG3Δ*env* and the Env complementation plasmid, pSVIII JRFLΔCT(+) as previously described at a ratio of 3:1. A 3:1 ratio of the transfection reagent, Fugene (Roche, Indianapolis, IN, USA), to DNA was used for transfection. Cell culture supernatants containing viruses were collected two days post-transfection. Neutralization capacity of the mAbs was determined using single-round infection by HIV-1 Env-pseudoviruses of TZM-bl target cells expressing CD4, CXCR4, and CCR5^53^,^54^. TZM-bl-based neutralization assays were performed with serial dilutions of antibody samples. Diluted samples were pre-incubated with virus (~150,000 relative light unit equivalents) for 1 hr 37 °C before addition of cells. Following 48 hr incubation, cells were lysed and luciferase activity was determined using a microtiter plate luminometer and BriteLite Plus Reagent (Perkin Elmer). Neutralization curves were fit by nar regression using a 5-parameter hill slope equation as previously described^30^. The 50% and 80% inhibitory concentrations (IC_50_ and IC_80_) were reported as the antibody concentrations required to inhibit infection by 50% and 80%, respectively.

## Additional Information

**How to cite this article**: Dai, K. *et al.* HIV-1 Vaccine-elicited Antibodies Reverted to Their Inferred Naive Germline Reveal Associations between Binding Affinity and *in vivo* Activation. *Sci. Rep.* **6**, 20987; doi: 10.1038/srep20987 (2016).

## Supplementary Material

Supplementary Information
