# Supplementary material for: HIV-1 Vaccine-elicited Antibodies Reverted to Their Inferred Naive Germline Reveal Associations between Binding Affinity and in vivo Activation
Source: Sci Rep. 2016 Feb 16;6:20987. doi: 10.1038/srep20987 (PMC4754655; doi:10.1038/srep20987)
Supplement: Supplementary Information [file srep20987-s1.pdf]

HIV-1 Vaccine-elicited Antibodies Reverted to Their Inferred Naive Germline Reveal Associations between Binding Affinity and *in vivo* Activation

Kaifan Dai[1,2*], Salar N Khan[1,2*], Yimeng Wang[2], Linling He[2], Javier Guenaga[1,2], Jidnyasa Ingale[1,2,3], Christopher Sundling[4], Sijy O'Dell[5], Krisha McKee[5], Ganesh Phad[4], Martin Corcoran[4], Richard Wilson[1,2], John R Mascola[5], Jiang Zhu[1,2,3], Yuxing Li[1,2], Gunilla B Karlsson Hedestam[4] and Richard T Wyatt [1,2,3#]

[1]IAVI Neutralizing Antibody Center at TSRI, La Jolla CA; [2]Department of Immunology and Microbial Science, [3]the Scripps CHAVI-ID, The Scripps Research Institute, La Jolla CA; [4]Department of Microbiology and Tumor Cell Biology, Karolinska Institutet, Stockholm SE; [5]Vaccine Research Center, Bethesda MD

*Contributed equally

[#]Corresponding author information: phone: 858 784 7676; email: wyatt@scripps.edu or rwyatt@iavi.org; 10550 N Torrey Pines Rd, IMM 28, La Jolla CA 92037

Supplementary Figures:

Name	Sequence (5' - 3')	
	Heavy chain	Light chain
	Forward	
GEBT383	CCTGTGGTTCTTCTTCCTCCTG	CTCCTCTCCTCCTCACTCTCC
GEBT391	TCACCATGAAGTTTGGGCTGAG	GCTCCTGCTGCTCTGGC
GEBT393	CCATGAAGTTTGGGCTGAG	GGTAAGGAAGGAGAACAGTAGGA
GEBT403	CCCCAGAGCACAGCACCTCA	TCCTCCTCACCCTTCTCATTC
GEBT404	GGCCTGAGCTGGGTTTTCC	TTAATTGTGTGGACATCTGTT
GEBT412	GGCTGAGCTGGGTTTTCCT	CCTGCTGCTCTGGCTCCC
GEBT414	CATTTCTGTCCTCCACCACGATG	ATGAGGGTCCCCGTTCAGC
	Reverse	
GEBT383	CTCCCTGCACCTGCTCCTG	GCCCTGTTCCTGATTCTGA
GEBT391	GAGGTTCCCTGGGGAACTGTC	GTCCCCTTCTACCGACACC
GEBT393	GTTCCCTGGGGAACTGTC	TTGGGATGCAGAGGTGAC
GEBT403	TAATATTTCCTATATAATT	GAAGGAAGCAGCAGCCACAGA
GEBT404	GATGACGTCCCACATCCTGACA	GACTTTCAAAATCTGACTGAGTTT
GEBT412	GAAGATGACGTCCCACATCC	CTTCTACCGACACCATCAGAG
GEBT414	TCCACCTCCCATGGTTAGGTCC	GGTGCAGAGGTGACTTCAT

Table S1 . PCR primers used for genomic sequence amplification

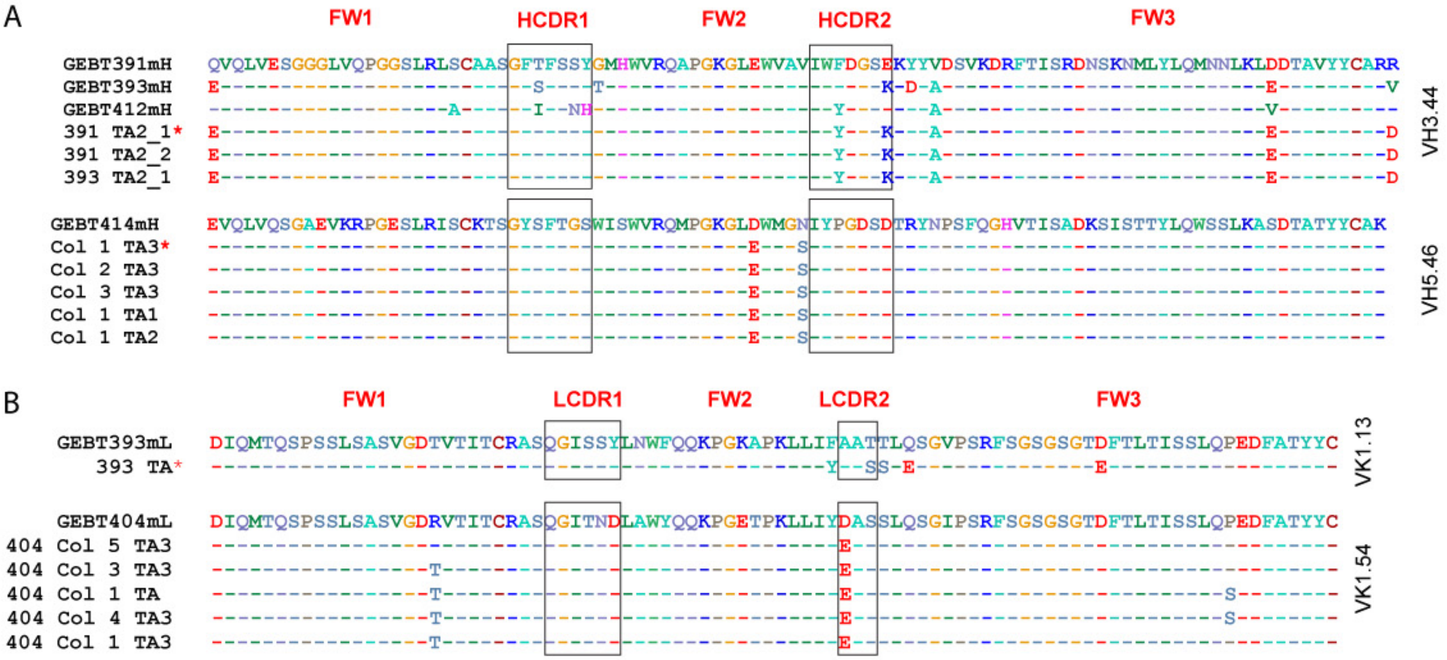

Figure S1 . Unique VH gene sequences (A) and VL gene sequences (B) obtained from NHP F128 T cell genomic DNA amplification and TA cloning. The first sequence in each gene family is the mature version and the sequences that follow are the TA clones. The closest germline genes as currently named in IMGT are shown on the right. The "*" indicates the clone's sequence which was used in the construction of the igL antibody. Sequences from 404 were analyzed but not used because the published database was the same except for one change and we decided to use it. Col - refers to colony.

Heavy chain amino acid alignment

```
                                               HCDR1                               HCDR2
GEBT383mH   QVQLQESGPAVVKPSETLSLTCAVSGGSISSSNWWSWIRQSPGKGLEWIGGVYGSSGSTEYNPS
GEBT383gH   -------------------------------------------------------I---G--------
GEBT391mH   QVQLVESGGGLVQPGGSLRLSCAASGFTFSS.YGMHWVRQAPGKGLEWVAVIWFDGSEKYYVDS
GEBT391gH   E-------------------------------.---------------------------Y---K---A--
GEBT393mH   EVQLVESGGGLVQPGGSLRLSCAASGFSFSS.YTMHWVRQAPGKGLEWVAVIWFDGSKKDYADS
GEBT393gH   ---------------------------T---.-G-------------------Y-----Y----
GEBT403mH   EVQLVQSGAEVKKPGSSVKVSCKASGYTFTD.YYMHWVRQAPGQGLEWMGEINPKTGGTNYAQK
GEBT403gH   Q------------------------------.--------------------------------
GEBT404mH   EVQLVESGGGLAKPGGSLRLSCAASGFTFSN.YWMYWVRQAPGKGLEWISAINSAGSRTYYPDS
GEBT404gH   -------------------------------.---H----------------------S---A--
GEBT412mH   QVQLVESGGGLVQPGGSLRLACAASGFIFSN.HGMHWVRQAPGKGLEWVAVIWYDGSEKYYADS
GEBT412gH   --------------------S------T--S.Y------------------------K------
GEBT414mH   EVQLVQSGAEVKRPGESLRISCKTSGYSFTG.SWISWVRQMPGKGLDWMGNIYPGDSDTRYNPS
GEBT414gH   -------------------------------.--------------E---S-------------

                                                        HCDR3
GEBT383mH   LKSRVTISIDTSKNQFSLKLSSVTAADTAVYYCVRHCTWYNNIWNGYYNGFDYWGQGVLVTSS   JH4
GEBT383gH   ---------------------------------------------------------------
GEBT391mH   VKDRFTISRDNSKNMLYLQMNNLKLDDTAVYYCARRGCTGSGCY.LYYYALDSWGQGVVVTSS   JH6
GEBT391gH   -------------------------E-------------------.------------------
GEBT393mH   VKDRFTISRDNSKNMLYLQMNNLKLEDTAVYYCARVNYN....W.NYVGGLDSWGQGVVVTSS   JH6
GEBT393gH   ---------------------------------------....-.------------------
GEBT403mH   FQGRVTMTRDTSTSTAYMELSSLRSEDTAIYSCARGG.QRLIP..LRRFYFYYWGQGVLVTSS   JH4
GEBT403gH   -----------------------------V-Y-----.-----..------------------
GEBT404mH   VKGRFTISRENAKNTLYLQMDSLRAEDTAVYYCAKGRIAIFG...LVITYFDYWGQGVLVTSS   JH4
GEBT404gH   ---------------------G------------------------...------------------
GEBT412mH   VKDRFTISRDNSKNMLYLQMNNLKLVDTAVYYCARRGCTGSGCY.LYYYGLDSWGQGVVVTSS   JH6
GEBT412gH   -------------------------E-------------------.------------------
GEBT414mH   FQGHVTISADKSISTTYLQWSSLKASDTATYYCAKGGGITIFG..LLHYPLDSWGQGVVVTSS   JH6
GEBT414gH   -------------------------------------------..------------------
```

Figure S2. Amino acid alignment of the antibodies. mH (mature heavy chain) is shown on top with the corresponding gH (germline heavy chain) compared below with only the changes highlighted. DH and JH regions are boxed in red and blue respectively.

Light chain amino acid alignment

```
                                                   LCDR1                LCDR2
GEBT383mL  QSALTQ.PPSVSGAPGQRVTISCTGSSSNIGGYYVSWYQQLPGTVPKLLIYENKKR
GEBT383gL  --V---.------D---------------------Y----F---A------D-N--
GEBT391mL  DIQMTQSPSSLSASVGDTVTITCRASQG..ISSYLNWFQQKPGKAPKLLIYAATTL
GEBT391gL  ----------------------------..-----------------------SS-
GEBT393mL  DIQMTQSPSSLSASVGDTVTITCRASQG..ISSYLNWFQQKPGKAPKLLIFAATTL
GEBT393gL  ----------------------------..--------------------Y--SS-
GEBT403mL  QSVLTQ.PPSVSGAPGQKVTISCTGSSSNIGGYDVHWYQQLPGMAPKLLIYDNNER
GEBT403gL  ------.-------------------------------------------------
GEBT404mL  DIQMTQSPSSLSASVGDRVTITCRASQG..ITNDLAWYQQKPGETPKLLIYDASSL
GEBT404gL  -----------------------Q----..---------------------E----
GEBT412mL  DIQMTQSPSSLSASVGDTVTITCRASQG..ISSHLNWFQQKPGKAPKLLIYAATTL
GEBT412gL  ----------------------------..---Y-------------------SS-
GEBT414mL  DIQMTQSPSSLSASVGDTVTITCRASQG..INNWLAWYQQKPGKAPKLLIYKASSL
GEBT414gL  --------A--------R-----Q----..-SS----------------L---PG-

                                                 LCDR3
GEBT383mL  PSGVSDRFSGSKSGTSASLTITGLQPGDEADYYCSAWDNSLSSHVFGSGTKLTVL  Jλ6
GEBT383gL  -------------------------------------------------------
GEBT391mL  QSGVPSRFSGSGSGTDFTLTISSLQPEDFATYYCLQHN..FYPYSFGQGTKVEIK  Jλ2
GEBT391gL  E--------------E----------------------..---------------
GEBT393mL  QSGVPSRFSGSGSGTDFTLTISSLQPEDFATYYCLQHN..FYPYSFGQGTKVEIK  Jλ2
GEBT393gL  E--------------E----------------------..---------------
GEBT403mL  PSGISDRFSGSKSGTSASLAITGLQTEDEADYYCQSYDSSMNAWVFGGGTRLTVL  Jλ2/3
GEBT403gL  -------------A-----------------------------------------
GEBT404mL  QSGIPSRFSGSGSGTDFTLTISSLQPEDFATYYCQHYY..STPPTFGGGTKVEIK  Jλ2
GEBT404gL  -------------------------S------------..---------------
GEBT412mL  QSGVPSRFSGSGSGTDFTLTISSLQPEDFATYYCLQHN..FYPYSFGQGTKVEIK  Jκ4
GEBT412gL  E--------------E----------------------..---------------
GEBT414mL  QSGVPSRFSGSGSGTDFTLTISSLQSEDFATYYCQQYN..SGPLTFGGGTKVEIK  Jκ4
GEBT414gL  ------M--------E---------P------------..---------------
```

Figure S3 . Amino acid alignment of the antibodies. mL (mature light chain) is shown on top with the corresponding gL (germline light chain) compared below with only the changes highlighted. JL regions are boxed blue.

A

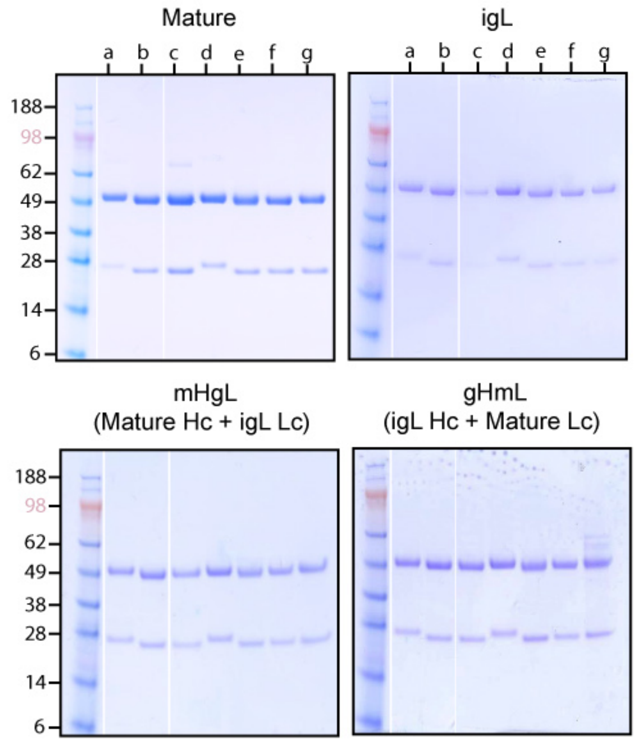

B

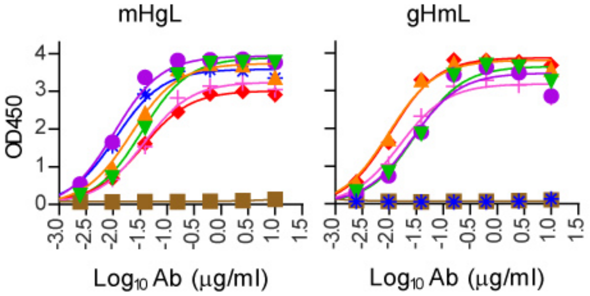

C

Post 2	igL LC	igL HC	igL
GEBT383	-	-	-
GEBT391	++	+	+
GEBT393	++	++	+
GEBT403	+	++	++
GEBT404	+	+	+
GEBT412	++	++	++
GEBT414	++	-	++

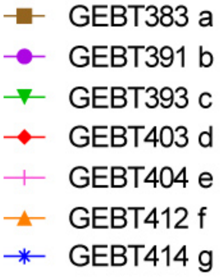

Figure S4. Reducing gels of all antibodies in the study and chimeric antibody binding to YU2 gp140-F. (A) Heavy and light chains of mature, igL and chimeric antibodies (a-g) on reducing gels. (B) Chimeric IgG binding to YU2 gp140-F by ELISA. (C) Chimeric antibody binding to gp140-F is depicted as a table.

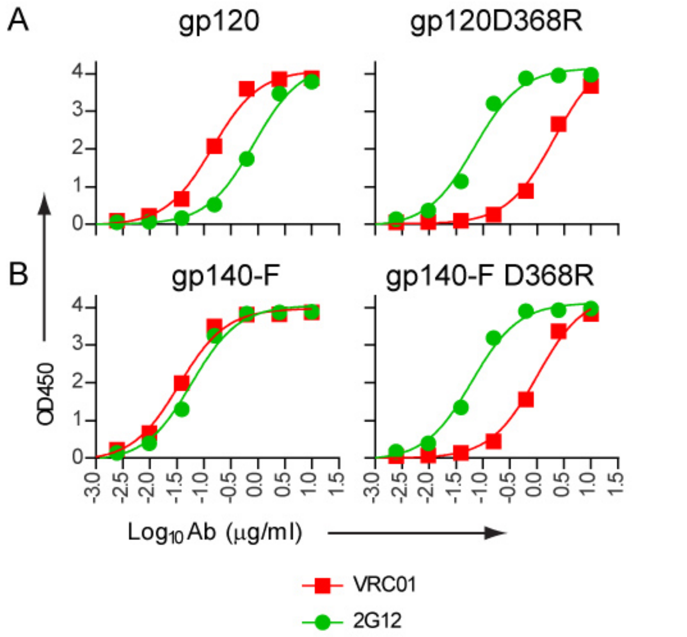

Figure S5. ELISA binding curves of the positive control antibodies. VRC01 and 2G12 antibodies binding to gp120 (A), gp140-F (B) and to the corresponding D368R mutants.

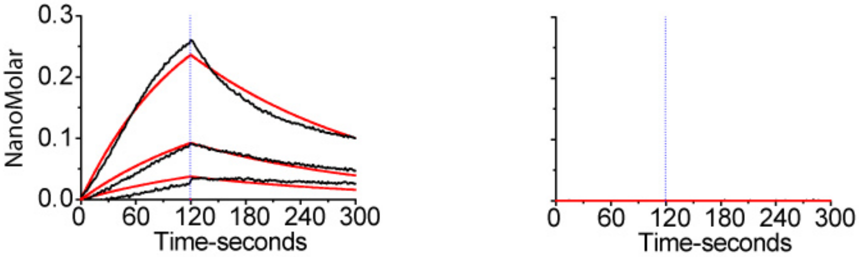

Figure S6. Binding analysis of GEBT 383.
GEBT383 antibody binds to His-Tag (left) and not to the control protein without the His-tag (right).

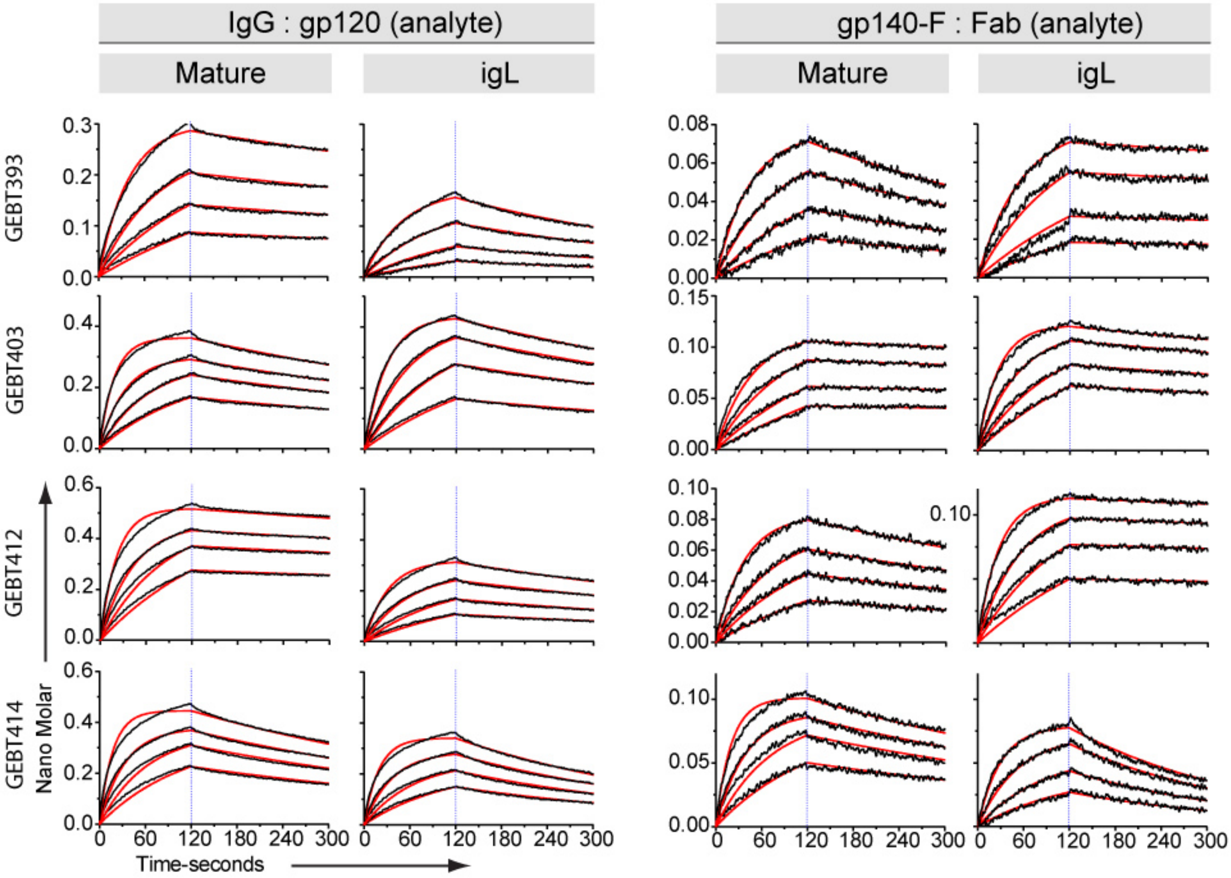

Figure S7. BLI binding curves of the antibodies. Mature and igL IgG binding to gp120 (two panels on left), and the same antibodies as Fabs binding to gp140-F (two panels on right).

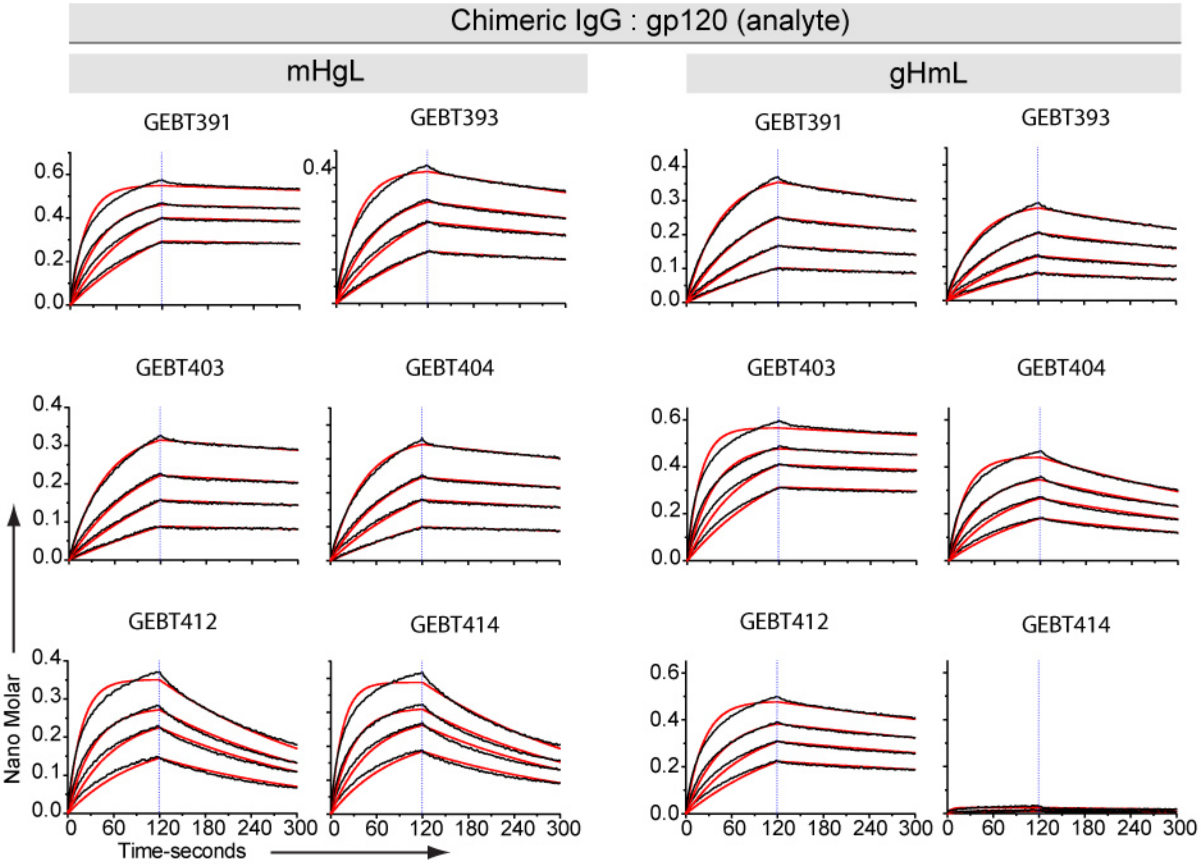

Figure S8. BLI binding curves of the antibodies. Chimeric IgG binding to gp120.
mHgL - mature heavy chain and igL light chain.
gHmL - igL heavy chain and mature light chain.

A

BLI	kD (nM)	kon(1/Ms)	kdis(1/s)		kD (nM)	kon(1/Ms)	kdis(1/s)
	IgG : gp120 (analyte)						
	igL				Mature		
GEBT391	118	1.72E+04	2.04E-03		6	9.21E+04	5.58E-04
GEBT393	146	1.76E+04	2.57E-03		15	5.74E+04	8.32E-04
GEBT403	11	1.34E+05	1.47E-03		13	1.14E+05	1.50E-03
GEBT404	56	4.95E+04	2.79E-03		43	9.08E+04	3.91E-03
GEBT412	104	2.84E+04	2.95E-03		11	8.38E+04	8.85E-04
GEBT414	63	4.86E+04	3.05E-03		24	1.13E+05	2.74E-03
	Fab : gp140-F (analyte)						
	igL				Mature		
GEBT391	86	1.78E+04	1.53E-03		2	1.61E+05	3.31E-04
GEBT393	101	2.11E+04	2.14E-03		5	7.07E+04	3.65E-04
GEBT403	2	1.71E+05	3.06E-04		5	1.16E+05	5.86E-04
GEBT404	32	9.85E+04	3.10E-03		7	1.22E+05	9.08E-04
GEBT412	55	2.61E+04	1.43E-03		2	1.47E+05	2.28E-04
GEBT414	36	1.20E+05	4.24E-03		9	1.94E+05	1.75E-03
	Chimeric IgG : gp120 (analyte)						
	gHmL				mHgL		
GEBT391	42	2.26E+04	9.47E-04		4	4.98E+04	2.08E-04
GEBT393	59	2.43E+04	1.42E-03		24	2.06E+04	4.96E-04
GEBT403	5	5.76E+04	3.12E-04		77	5.19E+04	4.01E-03
GEBT404	50	4.47E+04	2.23E-03		26	3.64E+04	9.59E-04
GEBT412	22	4.31E+04	9.35E-04		31	2.32E+04	7.12E-04
GEBT414	LB	-	-		61	6.33E+04	3.88E-03

B

SPR	kD (nM)	kon(1/Ms)	kdis(1/s)		kD (nM)	kon(1/Ms)	kdis(1/s)
	Fab : gp140-F (analyte)						
	igL				Mature		
GEBT391	30	9.53E+05	2.83E-02		27	6.19E+04	1.64E-03
GEBT393	43	9.09E+04	3.90E-03		12	2.06E+05	2.57E-03
GEBT403	2	2.57E+05	4.58E-04		7	2.28E+05	1.59E-03
GEBT404	17	1.23E+05	2.09E-03		7	2.62E+05	1.81E-03
GEBT412	16	1.77E+05	2.79E-03		2	2.84E+05	4.57E-03
GEBT414	9	1.34E+05	1.25E-03		6	1.21E+05	7.76E-04

Figure S9. Binding affinity (kD), association rates (kon) and dissociation rates (koff) of the antibodies. (A) IgG, Fabs and chimeric IgGs by BLI and (B) Fabs by SPR. The values shown in green are a repetition of data shown in Figure 3. LB - Low binding.

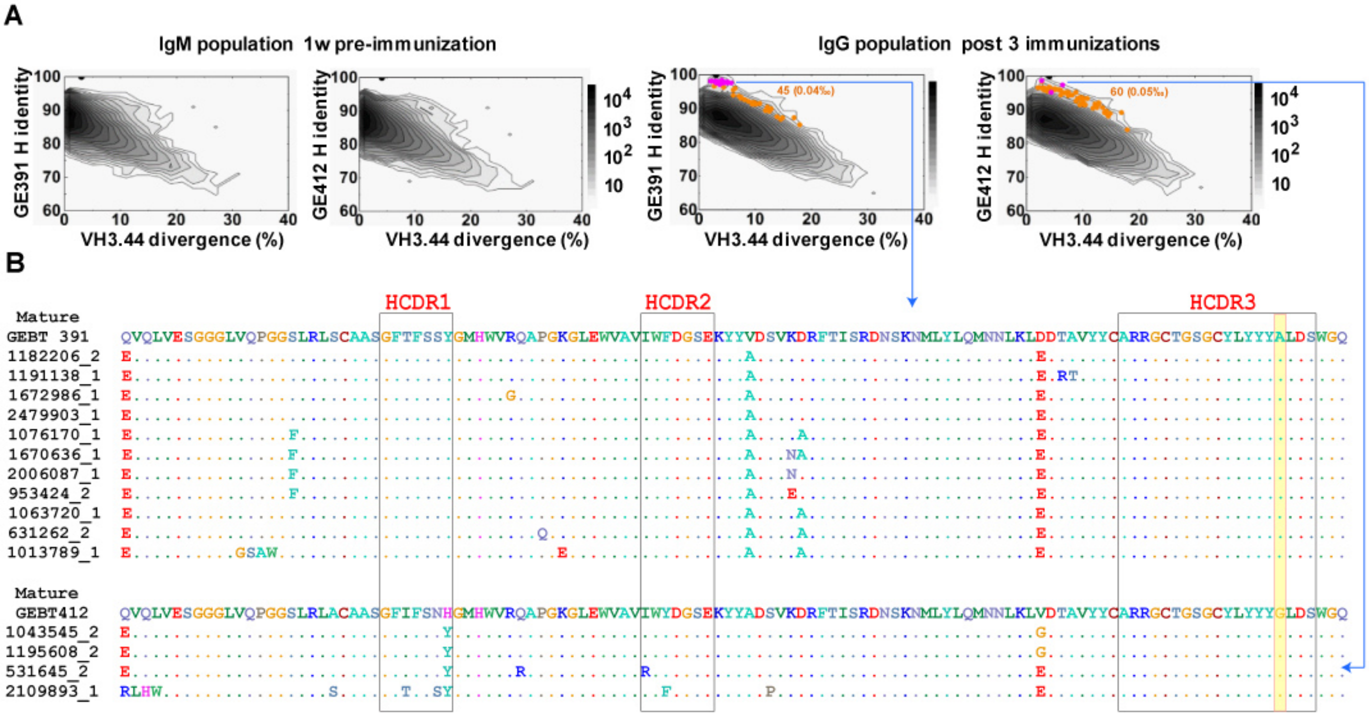

Figure S10: Germline gene usage of the VH chains derived from the unbiased antibody repertoire 3 weeks after the 3rd inoculation during F128 immunization. The distribution is plotted at the gene family level with the percent repertoire (%) labeled. (A) Identity/divergence analysis of heavy chain repertoires for CD4bs-directed mAbs isolated from F128 GE391 and GE412 is shown. The heavy chain population is plotted as a function of sequence identity to the template mAb and sequence divergence from putative germline V genes, with the template mAb labeled as a black dot on the plot partially visible at the top left corner of the plot. Color coding denotes sequence density. Sequences with an HCDR3 identity 95% or greater to the template mAb are shown in orange. The purple dots refer to matched sequences shown in B. (B) Alignments of sequences that show 100% HCDR3 identity to GEBT391 and GEBT412. The alignments were generated by the sequence alignment editor software, BioEdit.

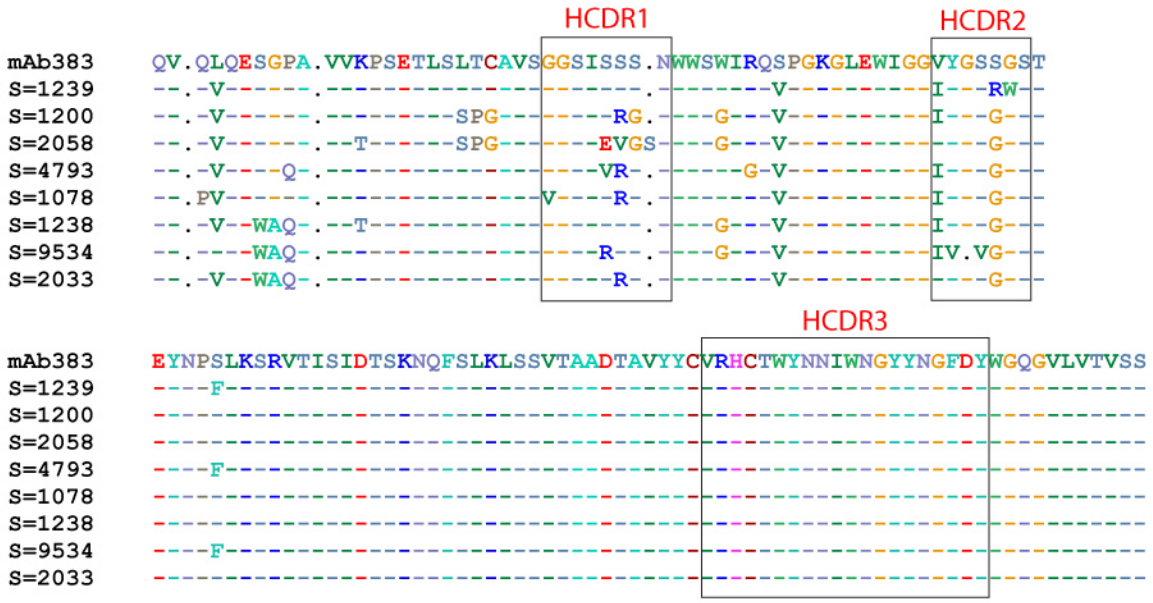

Figure S11. 5' RACE antibody lineage tracing of GEBT383. Selected sequences with HCDR3 identities 95% or greater to the template mAb GEBT383 mature HC are shown.

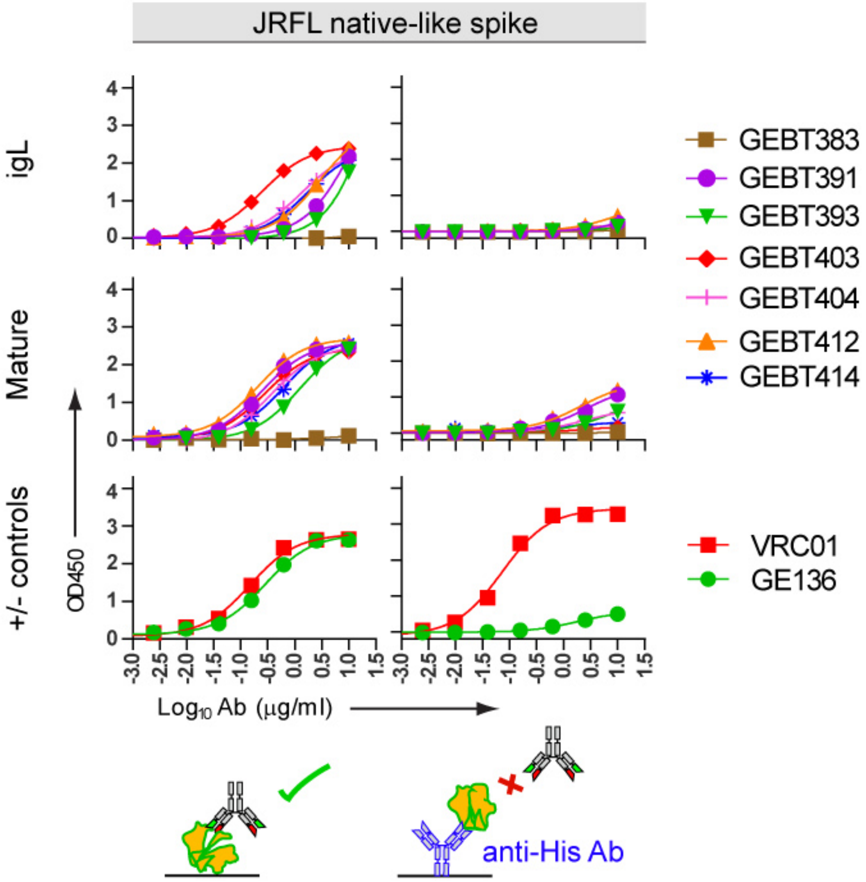

Figure S12. ELISA binding curves of igL and mature antibodies to JRFL-SOSIP native like spike that is either coated directly (left panels) or captured on a plate by anti-His antibody (right panels).

		Mature			igL	
		HxB2.DG.SG3	MN.3.SG3		HxB2.DG.SG4	MN.3.SG4
IgG	GEBT383	>50	15.1		>50	11.3
	GEBT391	1.36	0.223		>50	>50
	GEBT393	5.10	45.0		>50	>50
	GEBT403	0.455	0.269		0.216	0.211
	GEBT404	3.57	18.1		>50	17.5
	GEBT412	0.735	0.682		12.8	3.05
	GEBT414	>50	>50		>50	>50
Fab	GEBT383	>50	>50		>50	16.8
	GEBT391	0.924	0.004		>50	>50
	GEBT393	13.7	1.11		>50	>50
	GEBT403	0.931	1.20		0.997	>50
	GEBT404	5.53	0.469		>50	49.9
	GEBT412	4.51	>50		>50	>50
	GEBT414	>50	2.29		16.4	2.62
		mHgL			gHmL	
Chimeric IgG	GEBT383	>50	>50		>50	8.44
	GEBT391	0.669	0.256		>50	>50
	GEBT393	8.59	12.0		>50	>50
	GEBT403	0.380	0.320		0.510	>50
	GEBT404	6.86	>50		22.6	>50
	GEBT412	5.57	>50		14.0	0.668
	GEBT414	>50	>50		>50	13.0
+ve Control	VRC01	0.014	0.067			

Color shading represents potency as follows:

<0.100	0.100-1.00	1.00-10.0	>10.0

Figure S13. IC-50 neutralization values are shown for IgG, Fabs and Chimeric IgG.
